# Object Detection for UAV Aerial Scenarios Based on Vectorized IOU

**DOI:** 10.3390/s23063061

**Published:** 2023-03-13

**Authors:** Shun Lu, Hanyu Lu, Jun Dong, Shuang Wu

**Affiliations:** 1 College of Big Data and Information Engineering, Guizhou University, Guiyang 550025, China; 2 Bijie 5G Innovation and Application Research Institute, Guizhou University of Engineering Science, Bijie 551700, China; 3 Hefei Institutes of Physical Science, Chinese Academy of Sciences, Hefei 230031, China; 4 Anhui Zhongke Deji Intelligence Technology Co., Ltd., Hefei 230045, China

**Keywords:** object detection, UAV aerial images, VIOU loss, YOLOv5, multi-scale feature fusion network

## Abstract

Object detection in unmanned aerial vehicle (UAV) images is an extremely challenging task and involves problems such as multi-scale objects, a high proportion of small objects, and high overlap between objects. To address these issues, first, we design a Vectorized Intersection Over Union (VIOU) loss based on YOLOv5s. This loss uses the width and height of the bounding box as a vector to construct a cosine function that corresponds to the size of the box and the aspect ratio and directly compares the center point value of the box to improve the accuracy of the bounding box regression. Second, we propose a Progressive Feature Fusion Network (PFFN) that addresses the issue of insufficient semantic extraction of shallow features by Panet. This allows each node of the network to fuse semantic information from deep layers with features from the current layer, thus significantly improving the detection ability of small objects in multi-scale scenes. Finally, we propose an Asymmetric Decoupled (AD) head, which separates the classification network from the regression network and improves the classification and regression capabilities of the network. Our proposed method results in significant improvements on two benchmark datasets compared to YOLOv5s. On the VisDrone 2019 dataset, the performance increased by 9.7% from 34.9% to 44.6%, and on the DOTA dataset, the performance increased by 2.1%.

## 1. Introduction

**Figure 1:**
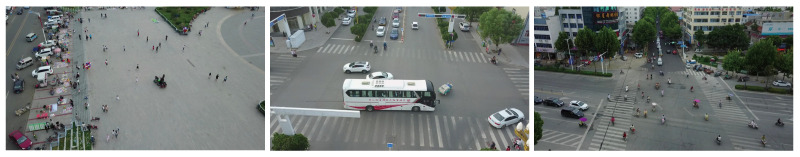
Object detection issues in UAV aerial images: a high proportion of small objects, multi-scale objects, a high overlap between objects, and complex backgrounds.

At present, there are two kinds of object detection methods used in aerial images that are both based on deep learning techniques. The first method is based on candidate regions and includes approaches such as R-CNN [1], Fast R-CNN [2], and Faster R-CNN [3]. The second method is based on regression and includes approaches such as the YOLO series [4,5,6,7,8,9,10,11], SSD [12], RetinaNet [13], and Centernet [14], as well as various other improved algorithms [15,16,17,18,19,20,21,22,23,24]. Object detection poses many challenges in unmanned aerial vehicle (UAV) images, as shown in Figure 1. The UAV aerial dataset contains a large number of small objects and the downsampling operation of the backbone network ignores a lot of useful information. Using features from the P3 of YOLOv5 [7] results in too many detailed features being discarded, which has a direct impact on the detection of small objects. There are a large number of objects with highly similar features in the dataset and overlapping objects pose additional challenges for the classification ability of the detection head. The original network uses a CIOU loss [25], which takes into account both the aspect ratio of the bounding box and the distance between the center of the real box and the predicted box; however, it only uses the aspect ratio as an influencing factor and its description of the width and height is vague. When the center points of the two boxes are consistent and their aspect ratio is the same but their width and height differ, the CIOU loss may not accurately reflect the actual object box. Additionally, the inverse trigonometric function used in the calculation can increase computational complexity.

To address the above-mentioned problems, in this paper, we propose a Vectorized Intersection Over Union (VIOU) loss to improve the regression accuracy of the bounding box. This loss uses several additional penalty terms to clarify the relevant factors involved in bounding box regression, such as the position (x, y) of the center point and the size and shape of the bounding box, which are beneficial for the direct regression of related parameters. For multi-scale objects, we explore how to fuse deep semantic features and shallow detail features to achieve the best detection results. Through four comparative experiments, we explore the sequence of fusion of shallow features and deep features, as well as the fusion method (incremental or decreasing), and propose a Progressive Feature Fusion Network (PFFN). Finally, combined with the decoupling ideas from YOLOv1 [4] and YOLO X [26], we propose an Asymmetric Decoupled (AD) head. We fully decouple the regression task from the classification task and use convolution kernels of different scales to provide the classification network with multi-scale feature information.

On the VisDrone 2019 dataset [27], the performance is improved by 9.7% from 34.9% to 44.6% compared to the original algorithm. On the DOTA dataset [15,28,29], the performance is improved by 2.1% compared to the original algorithm. In this paper, our contributions are as follows:

1. We propose the VIOU Loss, which simplifies the calculation and improves the regression accuracy of the bounding box.
2. We propose a new feature fusion network (PFFN), which fully integrates shallow features and deep features, addresses the issue of multi-scale objects, and improves the detection ability of small objects.
3. We propose an asymmetric decoupled head, which improves the network’s ability to classify and locate similar and overlapping objects.

## 2. Related Work

### 2.1. Regression Loss Function

In object detection, it is usually necessary to measure the overlap between the predicted box and the real box. In [30], the authors introduced the concept of the intersection-over-union (IOU) ratio, which divides the union of the two boxes by the intersection of the predicted box and the real box. The GIOU loss [31] introduces the minimum bounding box as a penalty item based on the IOU loss, which promotes the two boxes to keep getting closer and addresses the issue when the IOU is 0. The DIOU loss [25,32] introduces the Euclidean distance between the center points of the two boxes and the diagonal of the smallest enclosing box as indicators, which increases the convergence speed of the GIOU loss and addresses the issue that the two boxes cannot be effectively measured when they are surrounded by each other. Based on the above methods, the DIOU loss considers the coincidence of the center points of the two boxes and also includes the aspect ratio factor of the frame as a measurement index so that the prediction box can better complete the regression. The CIOU loss adds the loss of the detection box scale and the loss of the length and width based on the DIOU loss. However, the aspect ratio describes relative values, which can lead to ambiguity. The EIOU loss [33] calculates the difference between the width and height based on the CIOU loss to replace the aspect ratio and introduces the focal loss [13] to address the issue of unbalanced difficult and easy samples. The SIOU loss [34] redefines the penalty metric by taking into account the vector angle between the required regressions. The alpha-IOU loss [35] is a uniform exponentiation of existing loss functions based on the IOU loss.

### 2.2. Neck

The neck is designed to efficiently utilize the feature maps extracted by the backbone at different resolutions. Common object detection methods, such as Faster R-CNN, Mask R-CNN [36], YOLOv3 [5], RetinaNet [13], Cascade R-CNN [37], etc., use top-down unidirectional fusion FPN [38] to build an architecture with horizontal connections. By using low-level high-resolution detail feature information and high-level semantic feature information, they aim to achieve better predictions. Panet [39] is the first model to propose secondary fusion from bottom to top and is based on the FPN in Faster/Master/Cascade R-CNN, simply adding a bottom-up fusion path. Huang [40] proposed the Cross-Scale Feature Fusion of a Multi-Level Pyramid Network (CF2PN). DFF-PANet [41] can reuse feature maps in the backbone to enhance the detection capability of small- and medium-sized instances. Hilal Tayara [42] proposed a densely connected feature pyramid network through which high-level multi-scale semantic feature maps with high-quality information are prepared for object detection. Hong Tian [43] upgraded the existing FPN network output and improved the robustness of small target detection. In [44], the author studied the effect of re-merging three stage features for each stage based on the FPN of YOLOv3 [5]. The fusion of different stage features adopts the attention mechanism so that the contribution of other stages to the features can be controlled. NAS-FPN [45] is composed of top-down and bottom-up connections, which can fuse features across scales. The idea of Bi-FPN [46] is the same as that of NAS-FPN, that is, to find an effective block in the FPN and then repeat the superposition so that the size of the FPN can be freely controlled. Recursive-FPN [47] inputs the fused output of a traditional FPN to the backbone for a secondary feature cycle.

### 2.3. Detection Head

Mask R-CNN introduces an additional detection head for instance segmentation. IoU-Net [48] proposes a separate branch to predict the IOU loss between the box and the real box and learn the uncertainty of the bounding box prediction through an additional task to improve the localization results. YOLO X proposes a decoupled head, which uses two parallel branches (each branch includes two 3 × 3 convolutional layers) for regression and classification, respectively, and adds an IOU branch to the regression branch. Song et al. [46,49,50] proposed that in the localization and classification tasks of object detection, the focus and interest of the two tasks are different. Wu et al. [51,52,53] reinterpreted the two subtasks of classification and positioning in the detection task and found that the fc-head was more suitable for classification tasks and the conv-head was more suitable for positioning tasks. Therefore, it is inappropriate to integrate regression and classification tasks into one network. We propose a new asymmetric decoupled detection head, which separates the classification and regression tasks and improves the classification and localization capabilities of the network.

## 3. Methodology

**Figure 2:**
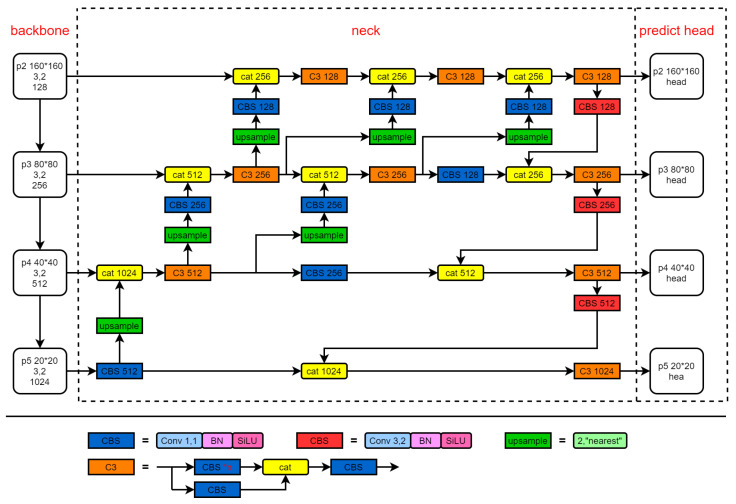
The architecture of the network: (1) The backbone adopts Cspdarknet53, (2) The neck uses PFFN, (3) The predict head uses the AD head. The specific structure of each module in the network is described below.

Based on the characteristics of UAV aerial images, we construct a new regression loss function, the VIOU. After fully exploring the characteristics of feature fusion, a new feature fusion network is adopted, which is the “neck” part of the dashed box in Figure 2. We apply the asymmetric decoupled head to the network, which is the “predict head” part of the dashed box in Figure 2.

### 3.1. VIOU Loss

**Figure 3:**
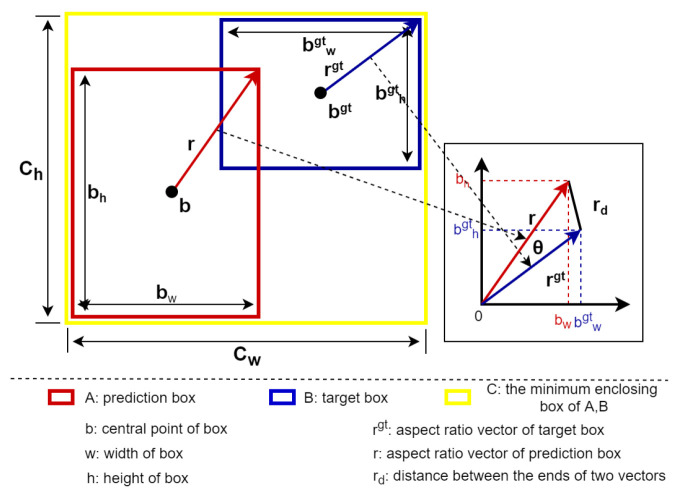
VIOU loss. The loss function is divided into three parts: the IOU loss, center point position loss, and vector loss of the width and height.

We continue with the IOU-based route and propose a more efficient version of the loss function, the VIOU loss, which is defined as follows:

(1) $$Loss_{V-IOU}=1-IOU+\frac{{\left(x-x_{gt}\right)}^{2}}{{\left(c_{w}\right)}^{2}}+\frac{{\left(y-y_{gt}\right)}^{2}}{{\left(c_{h}\right)}^{2}}+e^{-a*cos\theta }$$

where $c_{w}$ and $c_{h}$ are the width and height of the minimum enclosing box of the prediction box and the real box. $\frac{{\left(x-x_{gt}\right)}^{2}}{{\left(c_{w}\right)}^{2}}+\frac{{\left(y-y_{gt}\right)}^{2}}{{\left(c_{h}\right)}^{2}}$ represents the ratio of the difference between the horizontal and vertical coordinates to the width and height of the minimum enclosing bounding box, *a* represents the adjustable coefficient of the width and height of the penalty item, and the slope of the exponential function can be adjusted using the parameter *a*.

(2) $$cos\theta =\frac{r^{2}+{r^{gt}}^{2}-d^{2}}{2\times r\times r^{gt}}$$

where *r* and $r^{gt}$ represent the norm of the two bounding boxes, as shown in Figure 3. According to the cosine theorem, *d* represents the distance between the ends of the two vectors.

We divide the loss function into three parts: the IOU loss, center point position loss, and vector loss of the width and height, as shown in Formula (1). Due to the small coverage area of the small objects in the image, the regression of their bounding boxes is more challenging than large/medium-sized objects. In the prediction process, the prediction bounding box is offset by one pixel and the error impact on small objects is much higher than on large/medium-sized objects. The VIOU loss directly calculates the loss of the *x* and *y* coordinates of the center point of the boundary box instead of the distance loss between the two points, making it different from the current loss functions. The VIOU loss directly minimizes the difference between the center point locations (*x*, *y*) of the prediction box and the real box, making its regression more direct and resulting in better localization performance. At the same time, we use the width and height of the bounding box as a vector and utilize the translational invariance to make it share the origin of the coordinates to construct a triangle. The length of the two sides of the triangle is the norm of the vector constructed by the width and height values, as shown in Figure 3. The cosine function can express the relevant characteristics of the triangle, norms, and distance of the end of the two vectors, which directly constrain them in one formula and prevent divergence. By combining it with the exponential function to construct a composite function with parameter *a*, the proportion of the loss of the width and height can be adjusted. Through the cosine, the norm corresponds to the size of the bounding box and the angle of the vector corresponds to the aspect ratio of the bounding box. Thus, we can use the vector angle and vector norm to constrain the width and height of the bounding box. This is very helpful for the regression of small objects in multi-scale scenes. The VIOU loss can use a very simple formula to guide the regression of the position, shape, size, and other attributes of the bounding box.

### 3.2. Progressive Feature Fusion Network

In order to explore the best feature fusion method, we designed four new feature fusion networks, as shown in Figure 3. Since the proportion of small objects in the dataset is relatively high and the pixel size is small, we extract feature maps from the P2 of the backbone network to enrich the utilization of detailed features, and at the same time, add a P2 detection head of a 160 × 160 resolution, which will be responsible for small objects.

**Figure 4:**
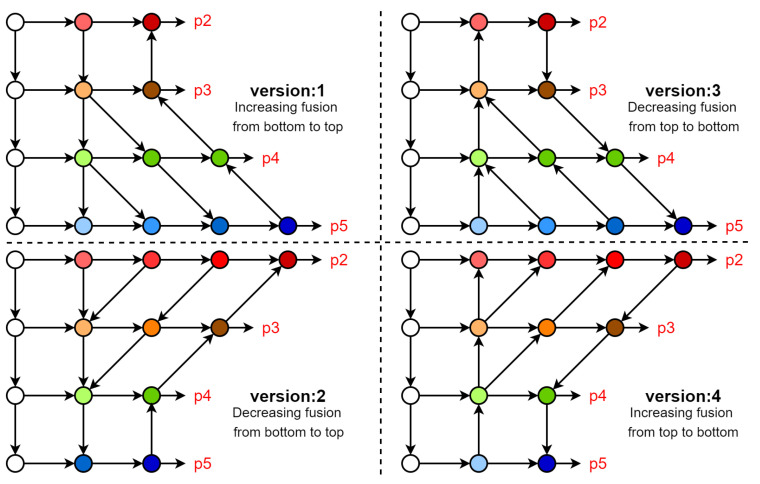
Structure diagram comparing the four groups of fusion networks. The specific modules are shown in Figure 2.

When using a convolutional network to extract image features, the first few layers of the backbone network can extract shallow features from the image, and as the network deepens, deeper features can be extracted. Shallow features have a higher resolution; contain more positional information, local information, and detailed information; and have fewer downsampling operations. Additionally, they are more friendly to small objects. Due to fewer convolutions, they have lower semantics and more noise. Deep features have stronger semantic information but have a low resolution and poor perception of details. If the two are efficiently integrated by taking their strengths and discarding their disadvantages, the model can be improved. To verify this using the above network, explore the best order of fusion of shallow features and deep features and explore the fusion method of increasing or decreasing, as shown in Figure 4. In our network, as the convolution deepens, each node will continuously fuse the features of its own layer with the features from the deep or shallow layers. In the process of fusion, the semantic depth of the shallow network is continuously deepened so that the deep and shallow features can be combined efficiently to achieve the best feature fusion performance. Each detection head uses divide and conquer to detect objects of corresponding scales from a local perspective and can also cover objects of different scales to the maximum extent from a global perspective to complete the detection task of multi-scale objects.

Through experiments, from the above four groups of networks, we finally chose version 4 as our feature fusion network, which we named the Progressive Feature Fusion Network. This network can continuously transfer deep features to shallow layers during the feature fusion process while avoiding the loss of detailed features caused by downsampling. Shallow detail features are fully combined with deep semantic features to achieve the purpose of complementary advantages.

### 3.3. Asymmetric Decoupled Head

**Figure 5:**
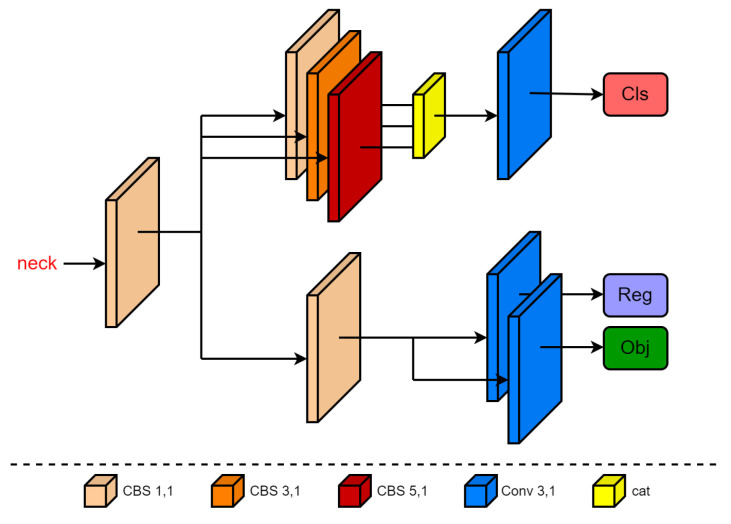
The structure of the asymmetric decoupled head. The specific details are indicated by different colors.

We improved the detection head of YOLOv5 and decoupled the two tasks of classification and regression, as shown in Figure 5. The decoupled head has an asymmetric structure and divides the feature map of the backbone network into two prediction branches after adjusting the number of channels using a basic convolution operation. In the classification branch, the feature map first passes the convolution operation with a convolution kernel size of 1 × 1, 3 × 3, and 5 × 5 and then splices the channels. The convolution layer of this branch provides different sizes of receptive fields for the input feature map, providing rich feature information for the subsequent classification and prediction tasks. In the regression branch, after the feature map is extracted by the 1 × 1 convolution, it is divided into two branches and the confidence prediction and box regression are performed by the 3 × 3 convolution. Compared with the classification branch, its regression branch uses fewer convolution modules, which reduces the calculations. The classification branch focuses on determining the category of the extracted features that are most similar to the object category, whereas the positioning branch concentrates on refining the center point coordinates, width, and height information of the box to correct the bounding box parameters. This makes the classification focus more on the central content and the regression focus more on the edge information.

## 4. Experiment

**Figure 6:**
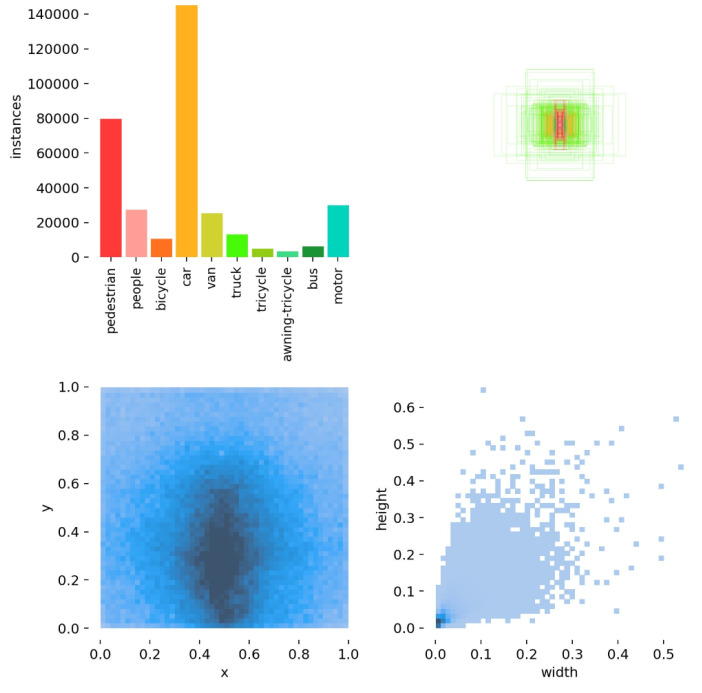
Information about the various types of objects in the dataset.

We chose VisDrone 2019-DET-train [27] as our training set and VisDrone 2019-DET-val [27] as our validation set. As can be seen in Figure 6, the dataset contained 10 categories, including “car”, “pedestrian”, and “motor”. It contained many confusing targets such as “pedestrian” and “people”, “bicycle” and “motor”, and “tricycle” and “awning-tricycle”. These were mostly small objects and most were located below the middle of the picture. During training, we set the model’s conf-thres to 0.5, IOU-thres to 0.45, and batch size to 8. To avoid overfitting or underfitting, we used mosaic enhancement and label smoothing and trained using 300 epochs. We used the SGD optimizer for training and used an initial learning rate of 0.001 with the cosine lr schedule. All models were trained on an NVIDIA RTX 3090 GPU. We chose YOLOv5s as the baseline and its corresponding weights were used for pre-training.

### 4.1. VIOU Property Comparison Experiment

**Figure 7:**
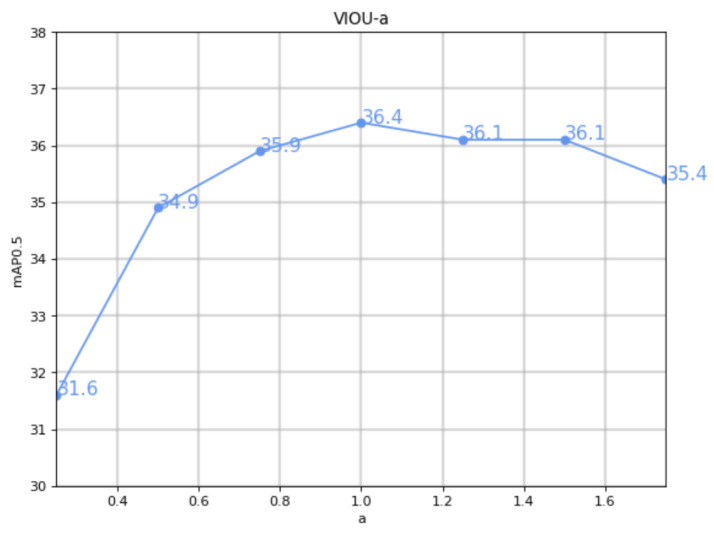
The graph of VIOU-*a*: the horizontal axis represents the value of *a* and the vertical axis represents the corresponding mAP0.5.

In order to achieve the best performance, parameter *a* was tested with 7 values ranging from 0.25 to 1.75 at intervals of 0.25. As shown in Figure 7, mAP0.5 gradually increased with the increase in *a*. When *a* was equal to 1, mAP0.5 reached a peak value of 0.364 and then gradually decreased. It can be concluded that for this dataset, the optimal value of *a* is 1.

We applied some of the current major regression loss functions to YOLOv5 on the VisDrone 2019 dataset and kept all the hyperparameters and other conditions. As can be seen in Table 1, when we used our proposed VIOU as the regression loss function, the best result achieved by the mAP0.5 was 36.4, which was an increase of 1.5% compared to the baseline (CIOU) and an increase of 0.7% compared to the other best loss function (alpha-IOU).

### 4.2. Comparison Experiment of Feature Fusion Characteristics of Neck Network

Four networks were designed to compare the mAP0.5 of Panet, specifically to explore the working characteristics of the feature fusion network. It can be seen in Table 2 that after introducing the features from P2 of the backbone into the network, the four feature fusion networks all performed well. After adding the P2 high-resolution detection head, the networks could focus on small objects and retain a large number of detailed features. Among them, the version 1 network had the worst performance as it employed incremental fusion from the shallow layer to the deep layer. Due to the insufficient mining of shallow feature information, it continued to downsample when merging with the deep layer, resulting in a loss of feature details from the shallow layer. Additionally, the deep layer contained relatively rich semantic information, making it difficult for the fusion to complement the advantages of both shallow and deep layers. The version 2 network adopted a decreasing fusion method from the shallow layer to the deep layer. In the same way as version 1, the features of the shallow layer were continuously downsampled and the features of the deep layer were fused. Therefore, due to the downsampling, the advantages were not complemented. However, the network was useful for the mining of shallow information so the small object information had relatively sufficient semantic information in the high-resolution layer. The version 3 network descended from deep to shallow fusion. This method was similar to that used in version 2. Although its shallow layer maintained the same convolution depth as version 1, it did not undergo downsampling to retain feature details, and at the same time, it incorporated rich semantic depth from the deep layers. Among the networks, the best performance was achieved by the version 4 network, which employed incremental fusion from the deep layer to the shallow layer. It utilized a method of deepening the convolution depth of the shallow layer feature map and transferring from the deep layer to the shallow layer to fuse the semantic information. This allowed each node to continuously integrate rich semantic information from the deep network, avoiding the loss of detailed features due to downsampling and preserving shallow geometric details.

### 4.3. Ablation Experiment

We experimented with each method on the VisDrone 2019 dataset and the results are shown in Table 3. The main evaluation indicators are accuracy, recall rate, mAP0.5, and mAP0.5:0.95.

***VIOU:*** The VIOU (a = 1) considered the IOU, the center point distance, and the shape and size of the box when the bounding box was regressed, which reduced the difficulty of the convergence of the regression box and the situation of wandering around during training. When we changed the original loss function of the network to the VIOU, its mAP0.5 increased by 1.5%, which had a good effect on helping the bounding box regression during training.

***PFFN:*** It can be seen from the data that when the PFFN was applied to the network, the above four indicators were significantly improved. This shows that the original feature fusion network did not fully mine and extract the features of the backbone network, especially when we made a large change to the shallow structure, and achieved good results. The original network was not friendly to objects with large-scale changes. Through the redesigned feature fusion network, a certain number of fusion convolution nodes were added at different resolution levels for semantic mining and the detailed features were retained. The rich semantic information from the deep layer was continuously fused and the fusion was fully compensated for. Insufficient semantic information in the shallow layer was eliminated and the loss of context information from the deep feature map was reduced.

***AD head:*** After applying the AD head to the network, all four evaluation indicators improved. Therefore, separating the classification network and the regression network improved the detection performance. The experimental structure demonstrated that the focus and points of interest of the two tasks of classification and regression were different so if the same network was used for classification and positioning, the performance would be bad. It is worthwhile improving the classification and regression capabilities of detection by our AD head.

### 4.4. Comparison of Different Detectors

To verify the effectiveness of the method in this paper, we selected some detectors for detection on the VisDrone 2019 dataset and trained 300 epochs. The experimental results are shown in Table 4. It can be seen that none of the current state-of-the-art detectors had a high mAP, which indicates that they did not perform well in UAV aerial object detection. As a result, the proposed YOLOv5s based on the VIOU loss, PFFN, and AD head achieved better results than SSD, RetinaNet, YOLOv3-v8, and Faster R-CNN, which were designed to detect UAV aerial objects.

To better evaluate the detection validity of the proposed methods, we conducted some comparative experiments on the DOTA dataset. There were 15749 training sets and 5297 verification sets in this dataset. It contained 15 categories of remote-sensing detection objects, including “plane”, “ship”, “storage-tank”, “baseball-diamond”, “tennis-court”, “basketball-court”, “ground-track-field”, “harbor, bridge”, “large-vehicle”, “small-vehicle”, “helicopter”, “roundabout”, “soccer-ball-field”, and “swimming-pool”. As can be seen in Table 5, our methods outperformed YOLOv5 and the latest YOLOv8 by 2.1% and 1.3%, respectively. Compared with other target detection methods, our method also had more advantages.

### 4.5. Visual Analysis

**Figure 8:**
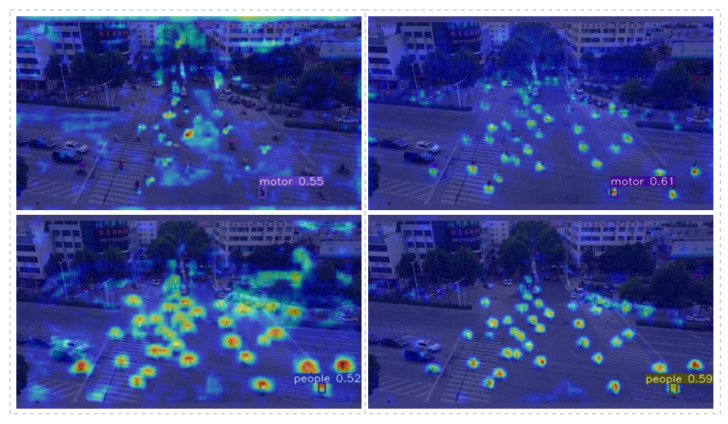
Image detection comparison heat map (the picture on the left is the result of direct detection by YOLOv5s, and the picture on the right is the result of our improved model detection). The darker the red color, the greater the value. The darker the blue, the smaller the value. Through the Grad-CAM, the probability value of the output category to be visualized is mapped to the last layer of feature maps and the gradient value of each pixel of the feature maps is obtained to determine how much influence each region has on the prediction of the model.

Figure 8 shows that the PFFN continuously fused feature information from deeper layers. The network achieved this by utilizing nodes with different resolution layers that allowed for the retention of a large number of detailed features from shallow layers. Additionally, the PFFN increased the convolution depth on each feature layer, avoiding the loss of small object features caused by downsampling. The added high-resolution detection head enabled the algorithm to better complete multi-scale detection tasks. The AD head could separate the classification and regression tasks, making the classification network more focused on the prediction of each object category and improving the network classification ability. A separate regression network makes its “points of interest” more focused on the coordinates of the center point of the object and the width and height of each object, making the border regression more precise.

It can be seen in the right heat map of the first group that we detected more overlapping small objects, which addressed the issue of losing detailed features due to high overlap between small objects and improved the detection of large object scale changes. In the right heat map of the second group, the thermal radiation of each “people” is more concentrated, resulting in a more accurate object frame position and improved regression prediction accuracy.

**Figure 9:**
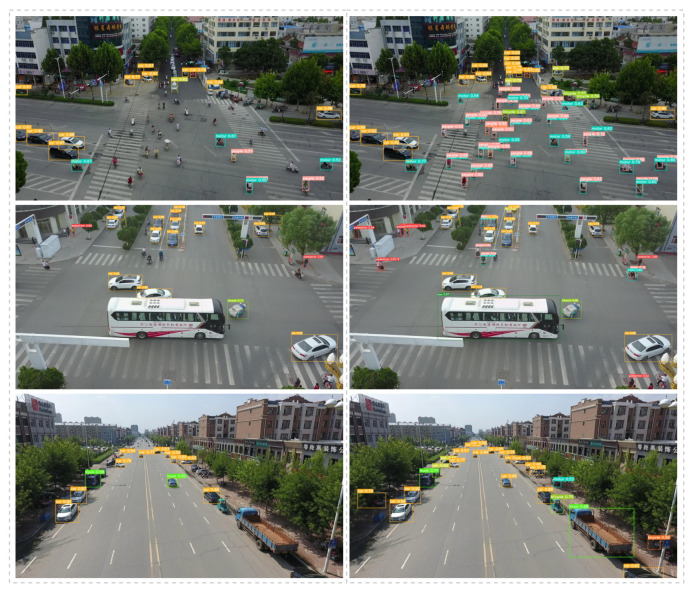
Picture detection effect comparison chart (the picture on the left is the result of direct detection by YOLOv5s and the picture on the right is the result of our improved model detection).

Figure 9 shows that there were some detection difficulties, which are common in UAV aerial images. In the first set of pictures, there are many “people” riding a “motor” and the object is small and highly overlapping. In the second group of pictures, the scale of the area occupied by the “bus” and “pedestrian” in the center of the picture is very different and the range of the object scale is too large. In the third group of pictures, some objects, e.g., “car” and “bicyle”, in the green belts on both sides could not be detected and the features are unclear or incomplete due to light or occlusion. It can be seen from the comparison that our methods achieved better performance.

**Figure 10:**
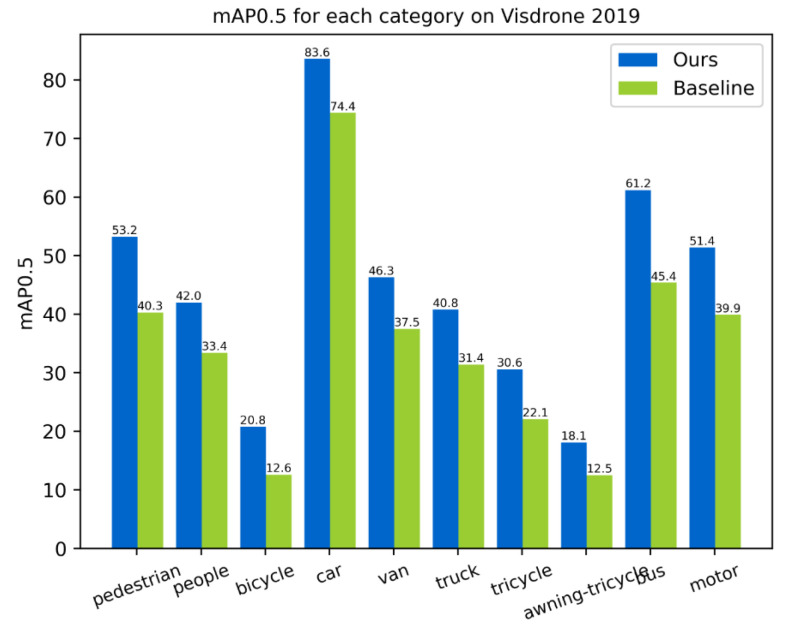
AP for each category on the VisDrone 2019 validation set.

The VisDrone 2019 dataset had 10 different categories. The mAP0.5 of each category after applying the VIOU, PFFN, and AD head on the validation set compared to the baseline are shown in Figure 10. It can be seen that the mAP0.5 improved across all categories with different sizes, which shows that our methods are appropriate for objects of various scales.

## 5. Conclusions

In this paper, our methods address the issues of multi-scale objects, a high proportion of small objects, and high overlap in UAV aerial images. The VIOU loss helps the regression of the bounding box during the training, making the positioning of the bounding box more precise. The proposed PFFN and AD head are used to allow the model to better adapt to the characteristics of UAV aerial image data to achieve the best detection performance. The PFFN reduces the loss of small object features caused by downsampling and at the same time, deepens the semantic depth of shallow features, greatly improves the detection ability of small objects, and improves the model’s ability to detect multi-scale objects. The proposed AD head is used to improve the regression ability of the network’s object box and object classification for overlapping objects. The experiment results show that the proposed model achieved an accuracy of 44.6%, which was 9.7% higher than the baseline and higher than other detectors. On the DOTA dataset, the performance was improved by 2.1% compared to YOLOv5s. Additionally, our methods are easily implementable, making them convenient to apply in practical scenarios.

## Figures and Tables

**Table 1:** Comparison of different metrics on the VisDrone 2019 dataset. (The bold type represents the best result).

Metrics	Precision	Recall	${\mathrm{AP}}_{0.5}$	${\mathrm{AP}}_{0.5:0.95}$
IOU [30]	48.2	34.6	35.0	19.3
GIOU [31]	46.4	34.4	34.3	19.1
DIOU [25]	47.1	34.0	34.6	19.2
CIOU [25]	48.4	34.6	34.9	20.5
SIOU [34]	46.9	34.4	34.5	19.0
EIOU [33]	46.7	35.5	35.5	19.5
alpha-IOU	48.1	35.8	35.7	20.5
VIOU(Ours)	50.9	34.9	**36.4**	**20.7**

**Table 2:** Performance comparison of the five feature fusion networks. (The bold type represents the best result. Pre: Precision; Rec: Recall; time: inference time (ms); memory: GPU memory (MiB)).

Neck	Pre	Rec	${\mathrm{mAP}}_{0.5}$	${\mathrm{mAP}}_{0.5:0.95}$	Time	Memory	Parameters
Panet [39]	46.0	26.9	34.9	20.5	1.5	2387	7,037,095
Version 1	48.6	38.3	38.8	22.2	2.1	2693	9,751,892
Version 2	50.5	39.3	40.0	23.1	2.4	2727	7,681,236
Version 3	52.1	40.0	40.5	22.9	2.0	2743	8,603,028
Version 4	**53.9**	**40.7**	**42.3**	**24.6**	2.2	2679	7,408,532

**Table 3:** Ablation study. (Pre: Precision; Rec: Recall; time: inference time (ms); memory: GPU memory (MiB)).

Version	Pre	Rec	${\mathrm{mAP}}_{0.5}$	${\mathrm{mAP}}_{0.5:0.95}$	Time	Memory	Parameters
baseline	48.1	34.6	34.9	19.1	1.5	2387	7,037,095
+VIOU	50.7	34.6	36.4	20.7	1.5	2387	7,037,095
+VIOU + PFFN	55.2	41.1	43.2	25.2	2.5	2657	7,408,532
+VIOU + PFFN + AD head	55.8	42.7	44.6	26.6	7.1	3805	19,258,068

**Table 4:** Comparison of performance on the VisDrone 2019 dataset. (The bold type represents the best result).

Method	Backbone	${\mathrm{mAP}}_{0.5}$	${\mathrm{mAP}}_{0.5:0.95}$
SSD [12]	ResNet-50	10.6	5.0
EfficientDet [46]	EfficientDet-D1	21.2	12.9
RetinaNet [13]	ResNet-50-FPN	25.5	15.1
CenterNet [14]	ResNet-50	29.0	14.0
Faster R-CNN [3]	ResNet-50-FPN	35.8	19.7
YOLOv3-SPP [5]	DarkNet53	18.9	10.6
YOLOv5	CSPDarkNet	34.9	19.1
YOLOv6 [8]	EfficientRep	28.8	19.0
YOLOv7 [10]	ELAN	37.5	23.8
YOLOv8 [11]	CSPDarkNet(C2f)	41.4	24.9
Ours	CSPDarkNet	**44.6**	**26.6**

**Table 5:** Comparison of performance on the DOTA dataset. (The bold type represents the best result).

Method	Backbone	${\mathrm{mAP}}_{0.5}$	${\mathrm{mAP}}_{0.5:0.95}$
SDD	VGG	42.7	23.1
EfficientDet	EfficientDet-D1	58.9	33.7
CenterNet	ResNet-50	56.7	30.8
Faster R-CNN	ResNet-50-FPN	62.9	30.4
YOLOv5	CSPDarkNet	71.4	45.9
YOLOv8	CSPDarkNet(C2f)	72.2	49.0
Ours	CSPDarkNet	**73.5**	**49.2**

## Data Availability

The code is available at https://github.com/jijiehao123/aerial-detection.git, and accessed on 10 January 2023.
